# Simultaneous Adalimumab and Antitubercular Treatment for Latent Tubercular Infection: An Experience from Nepal

**DOI:** 10.1155/2019/2034950

**Published:** 2019-04-01

**Authors:** Binit Vaidya, Shweta Nakarmi

**Affiliations:** Department of Rheumatology, National Center for Rheumatic Diseases (NCRD), 44600, Nepal

## Abstract

**Introduction:**

In Nepal, adalimumab is the most common agent being used, but in a disease activity-based dose tapering to address the economic constraints. Another constraint is the high risk of reactivation of tuberculosis in countries with high burden, especially with the use of tumor necrosis factor blocking agents. Though there are recommendations for screening and treatment of latent tuberculosis infection (LTBI) before using adalimumab, data is not clear regarding the appropriate screening schedule and the timing of initiation of biologic therapy.

**Methodology:**

This retrospective review of prospectively followed cohort of spondyloarthropathy patients aimed to evaluate the efficacy of simultaneous initiation of adalimumab with LTBI treatment. Patients fulfilling either the modified New York criteria for ankylosing spondylitis or Assessment in SpondyloArthritis international Society criteria and who were refractory to oral treatment were screened with Mantoux (≥10mm) and interferon gamma release assay (QuantiFERON) to detected LTBI. Those who tested positive were started on rifampicin/isoniazid combination for 3 months and adalimumab treatment on the same day. The patients were followed up at 2 weeks, 4 weeks, 12 weeks, and then every 3 months for 2 years.

**Results:**

Out of 784 patients diagnosed, 92 were receiving adalimumab. LTBI was detected by positivity of either Mantoux or QuantiFERON in 29.3% patients. None of the patients with LTBI who were started on the 2 drug regime simultaneous with adalimumab developed activation of tuberculosis. However, two patients testing negative for both the tests developed tubercular pleural effusion during treatment.

**Conclusions:**

Our findings indicate that screening for LTBI should be more frequent in patients from high tuberculosis burden countries; treatment of LTBI with rifampicin/isoniazid combination for 3 months is effective in preventing reactivation even when adalimumab is started simultaneously.

## 1. Introduction

Rheumatology is a growing subject in Nepal. With an increase in the diagnostic facilities and the availability of newer treatment options, an increasing number of patients are being considered for treatment with biological agents. Tuberculosis (TB) is one of the major concerns with the use of biological agents [1]. The risk is high mainly with tumor necrosis factor inhibitor (TNFi) like infliximab and adalimumab because of their inhibitory action on maintenance of granuloma [2]. In Nepal which is a country with high burden of tuberculosis, routine screening for latent tuberculosis infection (LTBI) is done before initiation of any TNFi treatment. Though the screening recommendations and treatment options for LTBI have been given by many authors and international guidelines, there is no consensus on the timing of first dose of biological agent after starting the treatment for LTBI [3]. Most authors recommend waiting for completion of treatment or at least one month after initiation of treatment for LTBI. But these are based on expert opinion, mostly from the pulmonologists. However, it is sometimes difficult to wait for long periods in patients with active diseases.

We studied the effectiveness of simultaneous initiation of LTBI treatment with first dose of adalimumab in preventing activation of tuberculosis in a cohort of spondyloarthropathy (SpA) patients.

## 2. Materials and Methods

### 2.1. Study Design

A retrospective analysis of the patients observed prospectively at National Center for Rheumatic Diseases (NCRD) in Kathmandu, Nepal, was performed. A comprehensive scheduled data was collected for each enrolled patient in a registry maintained at the center.

### 2.2. Patient Population

Patients aged ≥ 18 years diagnosed as either peripheral or axial SpA based on modified New York criteria or Assessment in SpondyloArthritis international Society (ASAS) criteria [4] were included in the study from July 2015 to July 2018. Purposive sampling method was done to select cases who were candidates for adalimumab. Patients with axial disease who were refractory to at least 2 nonsteroidal anti-inflammatory drugs (NSAIDs) at maximally tolerated dose over 4 weeks and those with peripheral disease refractory to combination of NSAIDs and 3 months of sulfasalazine at a dose of 50 mg/kg /day were started on adalimumab [5].

### 2.3. Latent TB Screening

Screening for LTBI was done with both tuberculin skin testing (TST) and interferon gamma release assay using the QuantiFERON TB gold method because all of our patients were vaccinated with BCG at birth. Plain X-ray chest was also done in all the patients at baseline along with detailed past history of tuberculosis infection and close contact with open TB cases. Either TST positive or IGRA positive was regarded as LTBI in this TST/IGRA combined method [6, 7]. Any doubtful finding was confirmed further with sputum examination and contrast enhanced chest computerized tomography. Sample for QuantiFERON assay was taken for each patient before administration of TST [3].

### 2.4. Tuberculin Skin Test (TST)

TST (augmented Mantoux test) was performed with 10 tuberculin units [8] of PPD- S. 0.1 ml was injected intradermally at the volar aspect of forearm and read after 72 hours by a single lab technician. A value of more than 10 mm was considered positive for patients on long term steroids or immunosuppressants. Use of sulfasalazine alone was not considered to be immunosuppressing.

### 2.5. Interferon Gamma Release Assay (IGRA)

QuantiFERON was performed using TB Platinum, Immunoshop. Whole blood was collected in Lithium heparin tubes and stimulated against* M. tuberculosis* specific antigens in the culture tubes to release interferon gamma (IFN-*γ*) which was assayed further. With the quantity of control IFN-*γ* < 400 pg/ml, a value of (test, control IFN-*γ*) ≥14 pg/ml and ≥ one-fourth of the control was considered positive. The value of (test, control IFN-*γ*) < 14 ng/ml or ≥14 pg/ml but < 1/4th of the control was considered negative.

### 2.6. Treatment for LTBI

Because Nepal is categorized as high TB burden country by the World Health Organization with increasing prevalence of multidrug resistance cases, we opted for combination therapy with rifampicin (10mg/kg) and isoniazid (5mg/kg) daily for 3 months as regimen of choice for treatment of LTBI [6].

### 2.7. Treatment of AS with Adalimumab

The patients fulfilling the criteria for AS and for the initiation of TNFi were started on adalimumab 40 mg subcutaneously. The first two doses were administered two weeks apart; then, a disease activity-based dose spacing was adopted to minimize the cost of therapy where the third dose was usually administered after 1 to 3 months and then every 4 to 6 months. All patients were receiving methotrexate at a dose of 7.5 to 15 mg per week along with folic acid 5 mg per week based on patient tolerance. Methotrexate was added with the first dose of adalimumab. In those patients testing positive for LTBI, adalimumab and rifampicin/isoniazid combination were started on same day.

### 2.8. Follow-Up Protocol

All patients (both groups receiving LTBI treatment and LTBI negative patients) were followed up at 2 weeks, 4 weeks, 12 weeks, and then every 3 months for 2 years. A single dedicated research officer recorded the demographic characteristics including age, gender, BMI, educational status, and HLA B27 (by polymerase chain reaction) status which were recorded at first visit. The disease activity scoring (Bath Ankylosing Spondylitis Disease Activity Index, BASDAI, and Ankylosing Spondylitis Disease Activity Score, ASDAS), inflammatory markers, baseline hemogram, and liver function tests were recorded at each visit. Two treating rheumatologists (authors BV and SN) decided on the subsequent dosing based on patient's assessment. They also assessed the patients for any symptoms and findings of tuberculosis reactivation at each visit and necessary investigations planned if indicated.

## 3. Results

A total of 784 patients diagnosed as either peripheral or axial SpA were maintained in the SpA registry at NCRD. Of them, 92 patients were receiving adalimumab for refractory disease. The mean age of the patients at presentation was 31.84 ± 12.49. Around 88.1% of patients were male (Table 1).

Eighty-four patients (90.9%) tested positive for HLAB27. The mean ASDAS CRP was 4.46 ± 1.28 with 74.4% having very high disease activity at baseline. Other clinical parameters are presented in Table 2.

LTBI was diagnosed (either Mantoux or QuantiFERON positive) in 27 (29.3%), among whom 6 were positive for both TST and IGRA, 15 were positive only for IGRA, and 6 were positive only for TST (Table 3). All patients had normal chest X-ray findings. Chest CT for screening for active tuberculosis was advised and the findings were normal. There were no cases of active tuberculosis or past history of tuberculosis in any patient.

Around 75% patients were receiving adalimumab at an approximate dosing of 40 mg every 4 months.

The average dose of methotrexate was 15 mg per week (IQR 7.5 to 20).

Tuberculosis developed in two patients during the follow-up. Both of them had tested negative for both Mantoux and QuantiFERON tests at baseline. Both cases presented with tubercular pleural effusion diagnosed on the basis of exudative pleural fluid with lymphocyte predominance and high levels of adenosine deaminase. One of the patients was a practicing clinician. The demographic and disease characteristics of patients developing tuberculosis are summarized in Table 4.

## 4. Discussion

### 4.1. Burden of Disease

Globally, TB is one of the most common communicable and fatal diseases especially in low- middle income countries [9] with the absolute number of TB deaths reaching 1.3 million in 2016 [10]. The total incidence of the disease was 10.2 million and the number of prevalent cases was 10.1 million in 2015 [9]. Nepal is a low income country [11] with higher rates of infectious and communicable diseases. According to annual report published in 2017, the burden of TB in Nepal is quite high with 120 new cases daily and 20 deaths per day [10]. In our study also, the prevalence of LTBI among SPA patients taking adalimumab was around 29.3%. In such situations, careful screening of TB and its treatment is mandatory in special situations like immunosuppressive therapy.

### 4.2. Risk of TB with Monoclonal Antibodies

Anti-TNF therapy is associated with higher rate of tuberculosis, especially with the monoclonal antibodies; adalimumab and infliximab [1, 12]. According to the Cochrane review in 2011, the odds ratio for reactivation of latent TB in patients was highest among certolizumab users (OR 4.43), followed by golimumab (OR 3.04), infliximab (OR 2.82), and adalimumab (OR 2.14). Risk of LTBI reactivation was low with etanercept (OR 1.48) and abatacept (OR 0.50) [13]. This can be explained by the fact that IFN *γ* and TNF *α* play an important role in inflammatory granuloma formation and its maintenance. Thus the use of anti-TNF therapies decreases the production of TNF*α*, eventually increasing the risk of reactivation of LTBI [2, 14].

During one year of follow-up period, two patients on adalimumab developed tubercular pleural effusion. Both of them tested negative for LTBI during screening. They were treated successfully with antitubercular therapy (ATT) category I. One of them restarted treatment with adalimumab after completion of ATT and is doing well. The other patient was switched to etanercept.

The low incidence of TB in our study may be attributed to lesser frequency of adalimumab, which eventually meant lesser dose. Adalimumab was given twice monthly, then dose spacing was done depending on disease activity. This dose spacing regime was introduced keeping the financial status of patients in mind, and it was well tolerated by the patients both physically and financially.

### 4.3. Subsequent Use of 2 Screening Tools

In this study, not all patients positive for IGRA tested positive for TST and vice versa. This indicated that performing only 1 test may result in false negative results, and higher rate of LTBI reactivation. For the patients at high risks or immunosuppression, a combination of history, physical examination, TST, IGRA, and CXR is the preferred modality of LTBI screening [15]. Some may use TST alone and other use IGRA alone for the screening purpose. Concurrent use of both screening tools is recommended by few especially in immunocompromised/immunosuppressed people [3, 15]. As the risk of reactivation of latent TB and new infection is higher in patients taking anti-TNF therapy, screening by IGRA may be done once a year in case of continuous immunosuppressive therapy [3, 15]. Similar result was seen in our study where 2 patients initially negative for LTBI developed new infection. This may be because of exposure to bacteria in such high burden country during treatment with adalimumab. So annual screening is justified in immunosuppressed situations.

### 4.4. Treatment

There are several regimens for treatment of LTBI, the common ones being isoniazid for 6-9 months; 3-month regime of weekly isoniazid and rifapentine; 3-4-month isoniazid plus rifampicin; 3-4-month rifampicin alone [3, 6, 16]. Isoniazid for 6-9 months is usually preferred treatment in low-middle income countries [6, 17] and also those receiving biologic therapy [2, 15]. A combination of rifampicin and isoniazid may also be used to prevent drug resistance especially in high TB burden areas [2]. We have followed the second regime of two drugs to reduce the risk of resistance. This regime was given to all 27 patients diagnosed with LTBI and it was well tolerated by the patients. No increased incidence of liver toxicity was observed during the treatment period.

### 4.5. Timing of Initiation of Biologic Therapy after Treatment of LTBI

The timing of initiation of biologic therapy after treatment of LTBI is still controversial [3]. The most abundant practice is starting biological agent after 1-2 months of initiation of ATT [2, 3, 18, 19]. However, earlier institution of immunosuppressive therapy may be considered after exclusion of active TB [15]. In this study, ATT for LTBI and adalimumab were initiated simultaneously. During the follow-up period of one year, none of the patients had reactivation of LTBI with such regime. This result shows that patient with high disease activity need not be treated with steroid or NSAIDs alone till we wait for 1 -2 months period. Simultaneous treatment with both the therapies is safe and beneficial. However, this result may also be due to less frequent adalimumab injections as per the protocol (dose tapering according to disease activity) used in the study. Though the usual dose of adalimumab in SPA patients is 40 mg subcutaneous every other week [20, 21], several studies have been conducted for tapering of biological therapies. The tapering may be done either by reducing the dose or by increasing the interval [22]. This study has tried tapering by increasing the interval between two doses. The interval was determined by disease activity of the patients measured by ASDAS and BASDAI. Such modification in treatment strategy made it affordable for most of the patients, and also the patients were exposed to lower dose of the biologic. It may be a reason behind safe use of ATT and biologic therapy in LTBI patients.

## 5. Conclusions

Tuberculosis infection rate is higher with the use of monoclonal antibodies, demanding meticulous screening for LTBI and its treatment. Simultaneous initiation of ATT and biologic therapy is safe in the patients, especially when the biologic therapy is used in lower dose.

## Figures and Tables

**Table 1:** Baseline demographic profile (n=92).

Parameters	Mean ± SD or n(%)	
Age	31.84 ± 12.49	

Gender	M, 81 (88.1)	
	F, 11 (11.9)	

Education	Can sign, 5 (5.4)	
	Primary, 15 (16.3)	
	Secondary, 43 (46.7)	
	Above secondary, 29 (31.6)	

Occupation	Housewife, 6 (6.5)	
	Student, 27 (29.3)	
	Office worker, 36 (39.2)	
	Business, 10 (10.9)	
	Others, 13 (14.1)	

BMI	24.53 ± 4.62	
Underweight	9 (10.3)	
Normal	24 (25.6)	
Overweight	14 (15.4)	
Obese I	38 (41.0)	
Obese II	7 (7.7)	

Joint pain	72 (78.6)	
Back pain	75 (81)	

Duration in months		Median
		(range)

Joint pain	72.30 ± 85.27	36.0 (1-360)

Back pain	71.09 ± 83.12	48.0 (1-360)

**Table 2:** Clinical and laboratory profile, N(%).

Parameters	N(%) or mean ± SD
Enthesitis	42 (45.2)

Red eye	34 (37.2)

IBD	2 (2.3)

Psoriasis	2 (2.3)

Fatigue	65 (70.3)

ESR	41.45 ± 26.51

CRP	61.71 ± 44.41

HLA B27	84 (90.9)

Fatigue VAS	5.30 ± 3.24

Spinal pain VAS	6.47 ± 3.01

Arthritis VAS	4.44 ± 3.86

Enthesitis VAS	2.73 ± 3.79

MS intensity	4.18 ± 3.41

MS duration	3.06 ± 3.88

ASDAS CRP	4.46 ± 1.28

ASDAS CRP	
Moderate	5 (5.1)
High	19 (20.5)
Very high	68 (74.4)

**Table 3:** MT and Quantiferon TB gold.

	IGRA positive	IGRA negative	Total
MT positive n	6	6	12

MT negative n	15	65	80

Total n	21	71	92

**Table 4:** Characteristics of patients developing tubercular pleural effusion (n=2).

	Patient 1	Patient 2
Age in years	23	29

Disease duration in months	96	22

HLA B27	Positive	Positive

TST at baseline	Negative	Negative

IGRA at baseline	Negative	Negative

ASDAS CRP at baseline	2.97	4.15

CXR	Right sided pleural effusion	Right sided pleural effusion

HRCT chest	Rt sided pleural effusion with mediastinal lymph node	

CRP mg/L	120	29.0

AFB	Not seen	Not seen

Pleural fluid		

TC per cumm	2960	4389

DC %	N 17, L 80	N 9, L 91

Protein gm%	5.7	4.9

Glucose mg%	79	56

ADA U/L	31.4	62.3

LDH	523	324

Culture	No growth	No growth

Adalimumab dose	6^th^ dose (6 months gap)	5^th^ (4 months gap)

## Data Availability

The data used to support the findings of this study are available from the corresponding author upon request.
